# Diagnostic Challenges in Patients with Inborn Errors of Immunity with Different Manifestations of Immune Dysregulation

**DOI:** 10.3390/jcm11144220

**Published:** 2022-07-20

**Authors:** Karolina Pieniawska-Śmiech, Gerard Pasternak, Aleksandra Lewandowicz-Uszyńska, Marek Jutel

**Affiliations:** 1 Department of Clinical Immunology, Wroclaw Medical University, 50-368 Wroclaw, Poland; 2 Department of Clinical Immunology and Paediatrics, Provincial Hospital J. Gromkowski, 51-149 Wroclaw, Poland; gerard.pasternak@umw.edu.pl (G.P.); aleksandra.lewandowicz-uszynska@umw.edu.pl (A.L.-U.); 3 3rd Department and Clinic of Paediatrics, Immunology and Rheumatology of Developmental Age, Wroclaw Medical University, 50-367 Wroclaw, Poland; 4 ALL-MED Medical Research Institute, 53-201 Wroclaw, Poland

**Keywords:** allergy, autoimmunity, autoimmune lymphoproliferative syndrome, inborn errors of immunity, lymphoproliferation, malignancy, primary immunodeficiency

## Abstract

Inborn errors of immunity (IEI), formerly known as primary immunodeficiency disorders (PIDs), are inherited disorders caused by damaging germline variants in single genes, which result in increased susceptibility to infections and in allergic, autoimmune, autoinflammatory, nonmalignant lymphoproliferative, and neoplastic conditions. Along with well-known warning signs of PID, attention should be paid to signs of immune dysregulation, which seem to be equally important to susceptibility to infection in defining IEI. The modern diagnostics of IEI offer a variety of approaches but with some problems. The aim of this review is to discuss the diagnostic challenges in IEI patients in the context of an immune dysregulation background.

## 1. Introduction

Inborn errors of immunity (IEI), formerly known as primary immunodeficiency disorders (PIDs), are inherited disorders caused by damaging germline variants in single genes, resulting not only in increased susceptibility to infections but also in allergic, autoimmune, autoinflammatory, nonmalignant lymphoproliferative, and malignant manifestations. According to the most recent report by the International Union of Immunological Societies (IUIS), the identified IEI were classified in 10 tables with subtables segregating groups of disorders into overlapping phenotypes: (1) immunodeficiencies affecting cellular and humoral immunity (combined immunodeficiencies); (2) combined immunodeficiencies with associated or syndromic features; (3) predominantly antibody deficiencies; (4) diseases of immune dysregulation; (5) congenital defects of phagocyte number or function; (6) defects in intrinsic and innate immunity; (7) autoinflammatory diseases; (8) complement deficiencies; (9) bone marrow failure disorders; and (10) phenocopies of IEI. The 55 novel monogenic gene defects positioned in the last IEI update enhanced the total number of IEI to 485 [1,2].

The COVID-19 pandemic had an impact on various fields of medicine. In the context of clinical immunology and IEI, it has uncovered several new IEI [1]. Each time, the appearance of new pathogens is a potential challenge for the general population and also healthcare systems because of the lack of significant pre-existing immune memory. Similarly, in the case of pathogens learned about so far, patients with specific germline genetic variants (causing known and unknown IEI) may be more exposed to severe disease than the general population. Research on the COVID-19 pandemic course led to the detection of genes and mechanisms necessary for anti-SARS-CoV-2 immunity. About 2–3% of cases of severe SARS-CoV2 infection resulted from germline LOF/LOE variants in the type 1 IFN signaling pathway: *TLR3*, *UNC93B1*, *TICAM1*, *TBK1*, *IRF3*, *IRF7*, *IFNAR1*, and *IFNAR2* [1]. According to Asano et al., X-linked recessive TLR7 deficiency is a highly penetrant genetic etiology of severe COVID-19 among 1.8% of males below the age of 60 years [3].

The defects of the number or the function of immune system elements determine the clinical presentation of an IEI. Family history, as well as personal and clinical data, are considered a core element of patient initial management. Extensive anamnesis and clinical evaluation are the main tools for a suspected diagnosis of IEI [4]. The early diagnosis of IEI can be life-saving but remains challenging due to the low prevalence of these pathologies. This can result in the delay of diagnosis and consequently in a worse prognosis [5].

Disease manifestation appearance (i.e., Nijmegen breakage syndrome (NBS), Shwachman-Diamond syndrome, and DiGeorge syndrome), as well as subject growth during both in utero life and later, may suggest the diagnosis of IEI and provide an important diagnostic clue [6]. Severe and/or recurrent infections, consanguinity, or an unexplained death in one’s family are well-known signs of IEI; however, more attention should be paid to signs of immune dysregulation. Immune dysregulation is defined as a breakdown or malfunction of molecular control of immune system processes, and it is used to characterize an array of autoimmune and inflammatory conditions [7]. According to IUIS classification, there are 10 IEI categories based on their underlying molecular defect. One of them is called ‘diseases of immune dysregulation’. Moreover, it has been established that other patients with humoral, cellular, or innate immune system deficiencies are also at risk of autoimmune or inflammatory conditions [8]. Currently, signs of immune dysregulation are of great importance in defining IEI, as well as an increased tendency to infection.

The modern diagnostics of IEI include various diagnostic measures, such as a simple blood count with particular attention paid to the total absolute lymphocyte count, the serum immunoglobulin levels, and the complete sequencing of the exome or genome [9]. However, during the clinical evaluation of a patient with suspected or confirmed IEI, we should be aware of the possible problems and finer points that may restrict diagnosis in patients with IEI. The aim of this review is to summarize these diagnostic challenges, in particular, in the context of immune dysregulation in IEI patients.

## 2. Allergic Disease

Allergy develops on account of disturbed function of the immune system. The immune system depends on a complex balance of activation, to defend against invasive, foreign pathogens, and control, to differentiate between self and foreign matter. Allergic reactions are exaggerated immune responses against specific allergens [10,11]. The comorbidity of IEI and allergy appears because of the impairment of the immune system, leading to infectious susceptibility; however, it is still able to trigger an allergic response [8]. The mechanisms underlying the relationship between atopy and immunodeficiency are better recognized, thanks to the discovery and characterization of genetic variants, often showing “a new face of old disorders” [8]. Several studies indicated the potential mechanisms leading to such dysregulation, which include the failure of central thymic tolerance, an imbalance between the effector and regulatory T-cell function, a failure in the production of counter regulating interferon-gamma (IFN-γ), disturbed cytokine production, and possible differences in microbial colonization and infection patterns [8,12,13].

Thanks to growing interest in the coexistence of allergy and IEI, the topic has been investigated in a number of studies. However, the results are still inconsistent. For example, in one Iranian study atopic dermatitis (AD) was present in 52% of patients with selective IgA deficiency (sIgAD) [14], while among Brazilian patients with sIgAD, AD was found in 2.3% [8,15]. In the USIDENT study, AD was most commonly reported in patients with a deficiency of the nuclear factor κB (NFkB) essential modulator (62.5%), the Wiskott–Aldrich syndrome (WAS: 41.5%), combined immunodeficiency (CID: 33.3%), selective IgM deficiency (33.3%), and autosomal-dominant hyper-IgE syndrome (AD-HIES; 25%) [8,16]. A cohort study of patients with early onset severe combined immunodeficiency due to adenosine deaminase deficiency (ADA-SCID) demonstrated that atopy was present in 56% of the patients, including mild AD in 11.1%. Severe AD was not a common feature [17]. A possible explanation of the diverse results are ethnic and geographical diversity and differences in methodological approaches.

Potential diagnostic difficulties may start even at the beginning in diagnosing IEI. An underlying, sometimes severe immune deficiency can manifest as common allergic symptoms, and IEI may masquerade allergic atopic patients [10]. In clinical practice, there are few warning signs of an underlying IEI among atopic phenotypes, and these include severe atopic disease, usually with a poor response to standard therapies, early-onset of the disease, a positive family history for IEI and/or severe familial atopy, and immunological abnormalities [11].

The standard screening tests for antibody deficiency include the measurement of immunoglobulin, IgG, IgA, and IgM levels in serum and the interpretation according to age-related reference values [18]. The routine measurement of serum IgE is not obligatory in the management of patients with suspected antibody deficiency and a history of recurrent infections. Previously, the level of total IgE was considered as a marker to catch allergic patients, but because it is nonspecific, it cannot confirm the allergy status of a patient [19,20]. Non-immunodeficient patients have variable IgE concentrations associated with atopic disease such as allergic rhinitis (AR), asthma, food allergy (FA), and AD, as well as other conditions, including parasitic disease [21]. However, in the context of PID, IgE measurement plays a role, especially in patients with concomitant eczema. Elevated IgE is common in a number of IEI, such as HIES, WAS, Netherton syndrome, immune dysregulation, polyendocrinopathy, enteropathy, X-linked (IPEX) syndrome, and Omenn syndrome [22]. One phenotype of complete DiGeorge syndrome, which is known as atypical complete DiGeorge syndrome, has oligoclonal T cell expansion with elevated IgE levels with concomitant generalized rash and lymphadenopathy [23]. The pathophysiological role of increased IgE in these disorders was not clearly characterized; however, there are few hypotheses [13]. Increased IgE production is associated not only with well-defined genetic syndromes but also with humoral, cellular, innate, and combined immunodeficiency disorders [5]. However, a high IgE (>180 IU/mL) is very rare in common variable immunodeficiency (CVID) (0.3% of patients) [21].

There are particular PIDs associated with atopy, especially eczema and elevated serum IgE, which can be confirmed by genetic tests and the identification of specific mutations. Mutations in the *WAS* gene on the X chromosome, which encodes the WAS protein (WASP), are a cause of Wiskott–Aldrich syndrome, characterized by recurrent infections, thrombocytopenia with small platelets, and eczema [8]. The mechanism for atopy in WAS is not fully described; however, impairment of regulatory T-cell (Treg) function is a possible contributor [8,24,25,26]. In total, 33% of patients with WAS and 20% of patients with X-linked thrombocytopenia (XLT) had positive food allergen-specific IgE (sIgE), in a study conducted by Lexmond et al. [8,27]. Food sensitization was generally detected with greater sensitivity using sIgE testing than by skin prick testing (SPT).

A dominant-negative heterozygous mutation in signal transduction and the activator of transcription 3 (STAT3) leads to autosomal-dominant hyper-IgE syndrome (AD-HIES), previously known as Job syndrome, with characteristic features such as chronic eczema, recurrent staphylococcal skin infections, pneumonia, increased serum IgE, and eosinophilia [10]. Skin findings distinguishing it from AD include a distinctive thickened texture of the facial skin, retroauricular fissures, and severe folliculitis of the axillae and groin [5]. Serum IgE levels are often >2000 IU/mL, and eosinophilia levels are often >700 cells/mL (eosinophilia does not correlate with the elevation in IgE), but patients usually do not suffer from symptomatic allergic disease such as AR, FA, or anaphylaxis [10,28]. Disturbances in the inflammatory process, and associated immune regulatory defects, are present. In clinical practice, a lower limit of 2000 IU/mL is often considered as a cutoff for AD-HIES. However, patients with HIES with lower IgE levels and STAT3 pathogenic variants have been reported [29]. Moreover, the serum IgE level does not correlate with the severity and activity of the disease, and paradoxically patients with STAT3 loss-of-function (LOF) mutations are rather protected from severe allergic reactions. A potential explanation of this protection is disturbed mast cell degranulation, as well as vascular reaction to histamine caused by the STAT3 mutation itself [8,30,31,32]. SPT results and clinical symptoms of allergy are consistent with the specific IgE (sIgE) results in AD-HIES. Both skin and blood test results are comparable between patients with AD-HIES and healthy controls [32]. Defective neutrophil chemotaxis has been described among AD-HIES patients, and variable specific antibody production is seen [5,33]. Patients may require immunoglobulin replacement.

At the end of 20th century, the National Institutes of Health HIES scoring system was originally presented where a score of 30 has a sensitivity of 87.5 percent and a specificity of 80.6 percent [34]. It is noteworthy that some patients (e.g., some young children), may not meet the scoring criteria. Thereupon, in cases of positive family history of HIES and some distinctive features, according to experts, molecular screening should still be performed even if the score is below 30. Other diagnostic guidelines takes into account five cardinal clinical features (recurrent pneumonia, newborn rash, pathologic bone fractures, characteristic facies, and high palate) with total IgE level and Th17 cell count [35]. Molecular genetic testing is crucial to establish the diagnosis of the AD-HIES.

Autosomal-recessive-HIES (AR-HIES) is characterized by highly elevated serum levels of IgE, eczema, recurrent staphylococcal abscesses, and hypereosinophilia. In contrast to AD-HIES, where patients are usually free from allergic manifestations, 50% to 70% of patients with AR-HIES suffer from severe allergies, i.e., eczema, anaphylaxis to food, and environmental allergies, and 30% have asthma [10,32]. Pulmonary disease is usually asthma-related as compared with AD-HIES, with pneumatocele and lung damage due to prior infections [10].

Some patients with DOCK8 or TYK2 deficiency were previously classified as AR-HIES with harmful allergic symptoms [36]. Now, we better recognize the differences in the clinical features. DOCK8 deficiency is a combined immunodeficiency characterized by allergic inflammation, severe atopy, high IgE, susceptibility towards cutaneous viral infections, and malignancy [37]. TYK2 deficiency is also a combined immunodeficiency with recurrent skin viral infections, while eczema and elevated IgE are variably found. A study conducted by Boos et al. revealed that total serum IgE levels similarly increased in STAT3-HIES, DOCK8 deficiency, and AD patients. The ratio of aeroallergen-specific IgE to total IgE was the highest in AD, whereas patients with DOCK8 deficiency showed the highest specific serum IgE against food allergens. Th2-cell numbers were significantly increased in DOCK8 deficiency and AD patients compared to STAT3-HIES patients and controls. The study showed that hyper-IgE syndromes and atopic dermatitis patients showed a different sensitization pattern of serum IgE corresponding to the allergic disease manifestations and Th-cell subset data, suggesting a key role of DOCK8 in the development of FA [32]. Moreover, according to Wilkie et al., defective Treg function may contribute to the increased skin inflammation and the eczema in DOCK8 deficient patients [38]. IEI with elevated IgE are summarized in Table 1.

On the other hand, low levels of IgE interest immunologists. Selective IgE deficiency (defined as a significant decrease in the levels of IgE (<2.5 IU/mL) in patients whose other immunoglobulin levels, including IgG, IgG subclasses, and IgA levels, are normal) has not been included in international classification systems for IEI [1]. Low serum levels of IgE can be associated with some well-defined IEI: common variable immunodeficiency (CVID), IgG subclass deficiencies, sIgAD, ataxia-telangiectasia (A-T), and agammaglobulinemia [39,40,41]. According to studies, an undetectable serum IgE (<2 IU/mL) occurs in only 3.3% of the general population [21]. In contrast, Lawrence et al. found that an undetectable IgE occurs in 75.6% of patients with CVID [21]. Another finding was a significant correlation between serum IgE with serum IgG, suggesting that lower IgE occurs in patients with more severe hypogammaglobulinemia. Moreover, false-negative results may appear using traditional methods of sIgE measurement, and allergen sIgE was not detectable in 96.5% of patients with CVID. Many patients with CVID report symptoms of rhinitis, wheeze, or adverse reactions to antibiotics, but it is difficult to detect allergic sensitization among them, especially using SPT or serum sIgE [41,42,43]. In these cases, sensitization should be confirmed using different methods, for example, an oral provocation challenge, and bronchial provocation tests with allergens [41]. The interpretation of food-specific IgE values and their usefulness in predicting symptomatic food allergies in the context of IEI patients is a potential field for further studies.

While diagnostics among PID patients during Ig replacement therapy (IRT) are often challenging, in the context of allergy, studies suggest that current Ig products are not a significant source of IgE [21].

## 3. Autoimmunity

There is also a high degree of overlap between autoimmune diseases and IEI in the context of genetic linkages and causes [44]. The molecular mechanisms responsible for the immune dysregulation in patients with IEI still are not fully recognized [45]. The usage of genetic analysis and a better understanding of the involved immune regulatory and signaling mechanisms is revealing the complex relationships between IEI syndromes and autoimmune diseases [44]. In the past, IEI and autoimmune diseases were considered as opposites; now, we know that genetic mutations may affect multiple immune cells and molecules, and in consequence IEI does not exclude autoimmunity. Furthermore, autoimmune diseases often coexist with some IEI [46].

The potential mechanisms associated with the pathogenesis of autoimmunity include impaired B cell differentiation and germ-center reactions, altered T cell central or peripheral tolerance, uncontrolled lymphocyte proliferation and differentiation, disturbances in Treg/Th17 balance, dysfunctional complement and innate immune activation, and the defective clearance of the infectious agents [45,46].

A French national study by Fischer et al. includes all types of IEI and autoimmune manifestations. The study demonstrated that autoimmunity is a significant component of clinical presentation of all types of IEI: one or more autoimmune and inflammatory manifestations were noted in 26.2% of 2183 retrospectively screened IEI patients, with a risk of onset throughout the patient’s lifetime. The risk of autoimmune cytopenia (AIC) was at least 120 times higher than in the general population; among children the risk of inflammatory bowel disease (IBD) was 80 times higher, while the risk of arthritis was 40 times higher. The risk of other autoimmune complications was approximately 10 times higher. Autoimmune manifestations occurred in patients with all types of IEI; however, patients with T-cell defects or CVID had, statistically speaking, the highest risk for autoimmunity [47].

The signs and symptoms of most rheumatic diseases are classified in international American College of Rheumatology (ACR) or European League Against Rheumatism (EULAR) criteria. The management of autoimmunity in patients with IEI is often challenging because immune dysregulation, as well as permanent inflammation, may influence the diagnostic process. Moreover, when assessing a patient with IEI for possible autoimmunity, it is important to consider a broad differential diagnosis, because infectious diseases, adverse effects of medications, and malignancies can mimic autoimmune processes. Thereupon, a complete diagnostic process is not effortless and requires a history, a complete physical examination, wide laboratory testing, imaging, and even pathological investigations [48]. Clinicians must be aware of the characteristic clinical features of autoimmune diseases among IEI patients. These include polyautoimmunity, which is defined as the presence of more than one autoimmune disease in a single patient and early onset autoimmunity (the presence of autoimmune disease at any age that is earlier than usual) [46]. Some IEI are associated with specific autoimmune diseases, and the awareness of these patterns also allows clinicians to monitor patients more effectively.

During evaluation of a patient with IEI and suspected autoimmunity, some laboratory tests are needed. This includes a complete blood count with differential, acute phase reactants, autoantibodies, serologies, flow cytometry, cytokine analysis, levels of complement, human leukocyte antigen (HLA) typing, and comprehensive endocrine and/or metabolic panels [48].

On the other hand, laboratory tests may help to catch patients with IEI among heterogenous group of patients with already diagnosed autoimmunity. Immune phenotyping and immunoglobulin (Ig) levels are indispensable. The ratio of naïve and memory T cells (CD45RA/CD45RO) may differentiate patients with late-onset or profound combined immunodeficiency disorders [49,50,51,52].

In addition, specific subsets of T and B cells have been linked to IEI with autoimmunity. These include the expansion of TCRαβ CD4^−^CD8^−^ (double-negative) T cells in autoimmune lymphoproliferative syndrome (ALPS), CD19^hi^21^lo^ B cells in CVID with autoimmunity, an abnormal count of Treg in Tregopathies, Th17 cells in STAT1 GOF patients, and expanding follicular helper T cells (Tfh) in CTLA4 and LRBA deficiency. Changes in these subsets may also predict the progression of autoimmune complications or a response to therapy [52,53].

Primary antibody deficiencies (PADs) are the most common inherited IEI in humans, with recurrent infections as a predominant presenting complaint. However, various types of PADs are also associated with inflammatory disorders, granulomatous lesions, lymphoproliferative diseases, and cancer. Several studies have reported that PAD patients are predisposed to autoimmune complications [47,54].

X-linked agammaglobulinemia (XLA), also known as Bruton agammaglobulinemia, is the prototype antibody deficiency [55]. Function-loss mutations in Bruton’s tyrosine kinase (BTK) lead to a block in B-cell maturation, a near total absence of B cells in the periphery, and severe reductions in serum immunoglobulins. Surprisingly, most patients with XLA have a small number of B cells, or “leaky B cells”, in the peripheral blood [54,56]. Patients with XLA are rather at a low risk of autoimmune or inflammatory diseases compared with other IEI patients, but several studies suggest that some XLA patients show symptoms with similar diagnostic features to rheumatoid arthritis (RA), IBD, alopecia, enteropathy, autoimmune hemolytic anemia (AIHA), immune thrombocytopenic purpura (ITP), neutropenia, and Kawasaki disease [54,57,58,59]. These patients are not expected to produce autoantibodies; however, surprisingly, the “leaky” production of autoantibodies and defects in B-cell central tolerance has been reported [54,60,61].

Autoimmune diseases occur in 20–30% of CVID patients. The most reportable are autoimmune cytopenias such as ITP, AIHA, and Evans syndrome; however, organ-specific and systemic autoimmune diseases are also described [45,62,63,64].

It is worth mentioning that it is not uncommon that autoimmune complications are the first or the only clinical manifestation of CVID during diagnostics [54,65].

A cohort study on CVID patients with immune cytopenia showed higher levels of serum immunoglobulin, CD19hi B cells, and T CD4 effector T cells, accompanied by reduced naïve T cells [45,66]. Moreover, according to several studies, Treg frequency and their functional characteristics are disturbed in CVID patients [54,67,68,69], which may result in elevated levels of activated T cells; autoimmunity; and chronic inflammation. Defects in Tregs are also correlated with the expansion of CD21low B cells in CVID patients with autoimmunity [70,71,72]. In a study by Boileau et al., the serum IgG level in CVID patients with autoimmunity (cytopenia and others) was greater than in CVID patients without autoimmunity [66]. Other studies revealed that CVID patients with autoimmunity have higher levels of IgM compared with non-autoimmune phenotypes [73,74]. On the other hand, markedly depressed serum immunoglobulin levels have been reported in patients with RA, Sjogren’s syndrome (SS), and systemic lupus erythematosus (SLE), prompting suspicion of IEI [75,76].

Autoantibodies circulating in the serum and/or plasma, as well as the immune complex deposits containing autoantibodies and complement, are essential diagnostic tools in most autoimmune diseases. In patients with hypogammaglobulinemia (i.e., CVID, XLA etc.) and some types of CIDs, diagnostic tests that are based on antibodies may be not useful and provide false-negative results. For example, the diagnosis of definite autoimmune hepatitis (AIH) in CVID patients is definitely challenging. According to the European Association for the Study of the Liver (EASL), both histologic evidence of moderate to severe interface hepatitis and the positivity of the typical autoantibodies are required to make an AIH diagnosis [77,78]. It is not surprising that CVID patients generally may not have autoantibodies, even in the case of noticeable autoimmune complications.

However, in a study by Tahiat et al. among 299 IEI patients with a dominance of PAD (27.8%) and CID (26.1%), autoantibodies were found in 32.4% of all IEI patients, compared with 15.8% of healthy subjects. Anti-nuclear antibodies (ANA) (10.0%), transglutaminase antibody (TGA) (8.4%), RBC antibodies (6.7%), anti-smooth muscle antibody (ASMA) (5.4%), and ASCA (5.0%) were the most common autoantibodies. The authors have concluded that considering the association of some autoimmune diseases with certain PIDs, screening for corresponding autoantibodies would be recommended. However, due to the low positive predictive value of the autoantibodies, the results should be interpreted with caution in patients with IEI [79].

Oppositely, the production of specific antibodies may by impaired even when the level of main classes of immunoglobulins is normal in specific antibody deficiency (SAD). Consequently, most autoantibodies are not found in these patients [48,80,81]. In sIgAD, as well as in CVID with IgA deficiency, it is obvious that there is a lack of antibodies in this immunoglobulin class (for example, tissue transglutaminase IgA–tTg IgA). On the other hand, among patients during IRT, exogenous Ig may interfere with some of the special immunologic tests. That is why it is worth considering if some screening tests such as autoantibodies should be performed before the therapy is being initiated or the serum should be frozen for future testing [48].

Some IEI patients are constantly negative for disease-specific autoantibodies, and in the case of clinical suspicion of autoimmune disease, other diagnostic methods should be considered. Medical imaging is often a part of the clinical evaluation of patients with suspected autoimmune disorder. In the case of IEI patients, some difficulties may appear at this point too. In particular types of IEI there is a problem with radiosensitivity, which limits the use of medical radiation for the diagnosis of autoimmunity [82,83,84]. Genetic instability, defective DNA repair, and a predisposition to malignancy are associated with specific types of IEI. A-T and NBS are well-defined IEI connected with defective DNA repair [85], where patients might be sensitive to radiation. X-ray exposure should be limited to diagnostic purposes only when it is medically necessary because patients should be protected from unnecessary medical techniques that incorporate radiation. Substitution with magnetic resonance imaging (MRI) or ultrasound is desirable [48].

Histopathological examination is sometimes crucial and clinically indicated in a diagnostic process. Diagnostic challenges may occur here as well. In IEI patients, as an effect of immunoglobulins and immune cells deficiency, affected tissue can have a different histological appearance in comparison to healthy individuals [48,78,86,87,88].

Since autoimmune cytopenia (AIC) is a common finding in IEI patients, Westermann-Clark et al. evaluated 154 pediatric patients with AIC in the context of IEI. Splenomegaly, short stature, and recurrent or chronic infections were common clinical features among patients with AIC and IEI. IEI patients were more likely to have AIHA or Evans syndrome than AIC-only patients. Patients with both IEI and AIC more often had low CD3 and CD8 cells; low IgA and IgG levels; and a higher prevalence of autoantibodies to red blood cells, platelets, or neutrophils. AIC diagnosis preceded IEI diagnosis by 3 years on average, except among those with partial DiGeorge syndrome [89]. The early detection of patients with comorbid IEI and AIC may improve treatment outcomes.

The main molecular defects and common autoimmune complications among IEI are summarized in Table 2.

## 4. Non-Malignant Lymphoproliferation

Ranging from reactive polyclonal hyperplasia (associated with immune disorders) to true monoclonal disease (malignant process), lymphoproliferative disorders (LPDs) constitute a heterogeneous group of diseases in clinical and genetic terms. LPDs occur when the physiological control of proliferation of both T and B cells collapses. Disturbances in this control may occur in many conditions where immunity is compromised. This creates difficulties (both in the clinical assessment of the patient and in the identification of pathogenic mechanisms) to differentiate LPDs [90]. They are observed in patients with immunodeficiency or immune dysregulation syndromes such as CVID, SCID, WAS, A-T, Chediak-Higashi syndrome (CHS), and X-linked lymphoproliferative disorders [91]. Additionally, splenomegaly and/or generalized lymphadenopathy are described in disorders such as CD27 deficiency, CD70, ITK deficiency, and XLP type 1. Autoimmune disorders, hypersensitivity reactions, and viral infections, including human immunodeficiency virus (HIV) infection, are also prone to developing lymphoproliferative disorders. Lymphoproliferation as well as lymphomas (both Hodgkin’s and non-Hodgkin’s lymphomas) are often associated with Epstein–Barr virus (EBV) infection. Moreover, both lymphadenopathy and splenomegaly can be caused by nonspecific infections, in CVID but also in almost any other PID, and they are not always primarily associated with immune dysregulation [92]. Transplant patients, as well as those taking immunosuppressants such as cyclosporine, sirolimus, and tacrolimus, are also at risk of developing benign LPDs [93].

Autoimmune lymphoproliferative syndrome (ALPS) is an example of a disease resulting from impaired apoptosis of lymphocytes, mostly as a consequence of abnormalities associated with programmed cell death mediated by Fas. Fas is a transmembrane receptor located on the cell surface and is one of the tumor necrosis factor receptors (TNFR). It is responsible for the induction of apoptosis, which is triggered after binding with the appropriate ligand (FasL). When the *FAS* gene is mutated, there are defects in the external pathway of programmed cell death [94]. Clinically, patients develop chronic lymphoproliferation and an increased number of T cells, which are referred to as “double negative T cells” (DNT) with CD4^−^/CD8^−^, CD3^+^, and TCRαβ^+^ phenotype [95].

ALPS usually presents in infancy or early childhood (the median age is 31–36 months), most often in the form of nonmalignant lymphoid expansion with lymphadenopathy, splenomegaly, and/or hepatomegaly and AIC, including hemolytic anemia and thrombocytopenia. In a minority of patients, clinical symptoms may appear later in life (18 to 35 years). In a French cohort, patients with later disease onset often presented autoimmune manifestations rather than LPD [96,97]. Patients often do not present symptoms that would suggest an infectious or neoplastic etiology. Most patients have an increased number of T and B lymphocytes, as well as polyclonal hypergammaglobulinemia. Hypogammaglobulinaemia, often not associated with increased susceptibility to infections, may occur in approximately 10% of cases. Autoimmunity is a common feature of ALPS and can be the first ALPS manifestation; however, it is not always present at the time of diagnosis. Autoantibodies are detected in up to 80% of patients, most often anticardiolipin antibodies or direct Coombs’ antibodies, but only half of them actually have an autoimmune disease, usually AIHA, ITP, or autoimmune neutropenia (AIN). A pledge of hemolysis during examination of blood smears, as well as the detection of autoantibodies and a degree of reticulocytosis, are helpful in distinguishing AIC from the effects of coexistent hypersplenism. Another helpful diagnostic tip is that AIC often manifests clinically. Autoantibodies typically have high affinity and are IgG-derived, in contrast to naturally occurring autoantibodies of the same specificity that are low-affinity and IgM-derived. Autoimmune diseases that affect other systems than the haematopoietic system can also occur but are much rarer [98]. Regardless of the time since the disease onset, symptoms such as lymphadenopathy and/or splenomegaly will ultimately be seen in 100% of ALPS patients and are required for diagnosis. The areas most commonly affected by lymphadenopathy are the neck, mediastinum, armpits, groin, and pelvis, although virtually any lymph node can become enlarged. Lymphoproliferation tends to subside over time, and by the age of 20, as much as 66% of patients achieve complete remission, while the rest of the patients experience a significant improvement. Infections are sporadic but can also occur as a result of neutropenia and/or nasopharyngeal obstruction due to lymphadenopathy [99]. Moreover, patients with ALPS are characterized by an increased risk of cancer (estimated at 10–20%); the most common forms of cancer are Hodgkin’s lymphoma and non-Hodgkin’s lymphoma [100].

Lymphoma can develop at any age in ALPS–FAS but is rare as a presenting feature. Distinguishing a benign node from a questionable node is a diagnostic challenge because of the frequent concomitant presence of benign/typical lymphadenopathy and splenomegaly seen with ALPS. Important clues for lymphoma are classic alarm symptoms (B symptoms), including fever, night sweats, itching, and weight loss. Positron emission tomography (PET)-based imaging may be helpful for distinguishing “good” from “bad” nodes on the basis of the presumed higher metabolic activity of malignant lymphoid tissue [101]. The nonmalignant lymphadenopathy fluctuates, and PET scan results fluctuate similarly. Lymphoma nodes more often are continuously chemically active (“hot”). Lymphoma typically originates in the B cell lineage, but T cell lymphomas have also occurred.

The required criteria for the diagnosis of ALPS include chronic lymphoproliferation lasting more than 6 months with the exclusion of neoplastic and infectious lymphoproliferation. In isolated lymphadenopathy, they must involve two distinct nodal regions. The second of the required criteria includes elevated counts of double negative T cells in peripheral blood that exceed 1.5% of the total number of lymphocytes or 2.5% in the case of T lymphocytes [102]. In addition, the diagnostics include genetic, biochemical (increased concentration of vitamin B12/IL-10/IL-18/sFASL/FAS), and histopathological tests.

## 5. Neoplastic Manifestations

Along with a predisposition to severe and recurrent infections and autoimmunity, neoplasms form a triad that identifies the most common symptoms in a variety of IEI. Despite this, there is a lack of systematic data on the cancer risk and type of neoplasms seen in most IEI. The development of malignant neoplasms most often occurs in patients with CVID, and in patients with defects in genes regulating DNA repair, cell cycle, apoptosis, or bone marrow maturation. Available population cohort studies suggest that the increased risk of developing cancer is limited to specific and rare forms of IEI and is mainly due to an increased risk of developing lymphoma [103,104,105,106]. The highest risk of lymphomas was reported in NBS (49%), X-linked lymphoproliferative syndrome (XLP; 24–30%), A-T (15–19%), ALPS (7–15%), and the mentioned CVID (1.8–8.2%) [96,103,107,108]. Among CVID patients, there is a 7- to 10-fold increase in gastric cancer incidence, which is related to the lack of secretory IgA [109,110]. In patients with CVID, extra-nodal non-Hodgkin’s B-cell lymphomas and mucosa-associated lymphomas are the most common [111]. Unlike most IEI, lymphomas in CVID are more common in people in the 4th to 7th decade of life and are usually EBV-negative [111,112]. In a study by Ludvigsson et al., individuals with IgA deficiency were at a moderately increased risk of cancer, with excess risks of gastrointestinal cancer. Children with IgA deficiency were at no increased risk of cancer, but the statistical power was limited in subanalyses [113].

Common high-grade DNA strand repair defects with chromosomal instability are seen in the A-T. Ruptures of dsDNA cause a high percentage of malignant tumors, chromosome instability, and abnormal rearrangements of V (D) J genes; a recombination of class switches and/or somatic hypermutations (the *ATM* gene in A-T, the *NBN* gene in NBS, the *DCLRE1C* gene in severe combined deficiency immunodeficiency with sensitivity to ionizing radiation and Omenn syndrome, the *LIG4* gene in the LIG4 syndrome, and the *LIG1* gene in DNA ligase 1 deficiency) cause complex immunodeficiencies and malignant neoplasms, most often lymphomas [114,115]. Patients with Bloom’s syndrome (*BLM* gene) age prematurely and are susceptible to non-Hodgkin’s lymphoma (NHL). Patients with Schimke syndrome (*SMARCAL1* gene) show chromosomal instability and an increased risk of malignant neoplasm, including NHL and osteosarcoma [116,117].

Malignancies associated with impaired telomere maintenance are observed in genetically heterogeneous congenital dyskeratosis and its clinically severe variant of Hoyeraal Hreidarsson syndrome, NBS and A-T. Disorders of telomerase lead to the defective function of rapidly dividing cells and increased susceptibility to hematological and solid tumors [114].

IEI, which inherently affect hematopoiesis, make it susceptible to malignant neoplasms. In Fanconi anemia, a genetically heterogeneous disorder, pancytopenia, hematologic malignancies, solid tumors, and clinical immunodeficiency phenotypes are observed. Mutations of the *WAS* gene coding for the WASP disrupt the connection between GTPases and the actin cytoskeleton, thus disrupting the regulation of signaling in hematopoietic cells. Myelodysplasia, leukemias, and lymphomas in patients with WAS are seen more frequently [107,114,118]. The deficiency of the hematologic transcription factor GATA2 leads to phenotypically variable immunodeficiency, primary alveolar proteinosis, Emberger syndrome with lymphedema and/or a predisposition to myelodysplastic syndrome, acute myeloid leukemia (AML), chronic myelomonocytic leukemia (CMML), and EBV lymphoma [119]. The risk of leukemia is increased with some severe congenital neutropenia (ELANE, HAX1, and WASP) but not increased with the *ELANE* mutation that causes cyclic neutropenia. An increased risk of leukemia has not been reported in other PIDs associated with neutropenia [120]. Mutations in the *CD40L* gene cause X-linked immunodeficiency with hyperimmunoglobulin M. In the case of CD40L and CD40 ligand deficiencies, a Cryptosporidium biliary tract infection may lead to sclerosing cholangitis, cirrhosis, and an increased risk of hepatocellular carcinoma and biliary tract cancer [121,122,123].

Almost 20% of all human malignancies are associated with chronic infections with such pathogens as HBV, HCV, HPV, EBV, HHV8/KSHV, HTLV-I, HIV-1, HIV-2, JCV, Merkel cell carcinoma (MCV), Helicobacter pylori, schistosomes, or hepatic flukes [124,125]. Additionally, in IEI patients, chronic infections are often associated with malignancies. They were mostly described in connection with EBV, HPV, and HHV8 infections [107,126,127,128]. HPV can cause cancer of the cervix, vagina, vulva, anus, and penis, as well as squamous cell carcinoma of the oral cavity. Patients with warts, hypogammaglobulinemia, infections, and myelokathexis (WHIM) syndrome are particularly prone to HPV infection, resulting in numerous warts, condylomata acuminate, and subsequent severe papillomatosis and malignant transformation of the lesions [128].

EBV in patients with IEI may cause chronic EBV viremia, hemophagocytic lymphohistiocytosis (HLH), dysgammaglobulinemia, atypical EBV-associated lymphoproliferative disorders (polymorphic B-cell hyperplasia, plasmocytic hyperplasia), and EBV-associated lymphomas [105,129,130]. In the rare heterogeneous KID syndrome (keratitis, ichthyosis, and deafness), mainly caused by mutations in the connexin 26 (*GJB2*) gene, 15% of patients develop squamous cell carcinoma, often in sun-exposed areas [131,132].

The estimated risk for developing cancer in patients with IEI ranges from 4 to 25 percent [133]. Furthermore, the diagnosis of the malignancy, both clinical and histological, can be challenging in the presence of non-malignant lymphoproliferation or bone marrow abnormalities. These states, as well as concomitant infections or complex co-morbidities, all can mimic a developing malignancy clinically, radiologically, and even histopathologically. Due to the statistically higher risk of the above-mentioned types of neoplasms, patients with IEI should undergo periodic age-appropriate screening tests, just like healthy people. However, the guidelines in this regard may differ depending on the IEI type and national or international recommendations. Patients with epidermodysplasia verruciformis (EV) should undergo regular dermatological check-ups due to an increased risk of skin cancer. Patients with A-T and their female family members with heterozygous mutant *ATM* should start the screening for breast cancer earlier than the general population, and this age depends on the type of the mutation in the *ATM* gene [134,135].

It is also worth mentioning that both NHL and Hodgkin lymphoma are diagnosed at younger ages in patients with IEI, and NHL is more common in males with IEI [136,137]. In patients with suspected lymphoma, medical management is the same as in immunocompetent patients; however, diagnostic difficulties may appear. Diagnostic tests useful in cancer screening include uric acid, lactate dehydrogenase (LDH), and erythrocyte sedimentation rate (ESR). Even histopathology, which is a gold standard of diagnosing malignancy, can be challenging in patients with IEI, particularly during the investigation of possible lymphoid malignancy.

If clinically indicated, a surgical biopsy providing sufficient material for the assessment of tissue architecture and ancillary diagnostic techniques is a better diagnostic option than needle core biopsy. Histological diagnosis may be difficult even when appropriate, high-quality material is gained [137,138]. For example, non-malignant lymphoproliferative lesions may precede, as well as co-exist with, lymphoid malignancies. Often, diagnostic boundaries between non-neoplastic and neoplastic lesions are ill-defined and difficult to apply. Lymphocyte clonality assessed by molecular techniques may help during diagnostics, but these alone cannot provide diagnostic certainty, and clonal B-cell and T-cell proliferations falling short of malignancy are not uncommon in IEI [138,139].

Patients with specific immunodeficiencies, including A-T, NBS, and CVID, should be informed about the increased risk of neoplasia associated with increased sensitivity to ionizing radiation. Before performing tests or therapy with the use of radiation, they should consult this fact with the attending immunologist. On the other hand, medical personnel should consider the benefit–risk ratio in terms of interventions with the use of ionizing radiation in the context of the underlying disease, taking into account the need to perform the examination, and the possibility of replacing the examination with radiation with alternative techniques without the use of ionizing radiation.

Advances in the diagnosis and treatment of patients with IEI contributed to a significant extension of the life of those patients who previously had no chance to live to adulthood. Patients with IEI require multidisciplinary care; therefore, physicians of various specialties should be aware of the increased tendency to develop neoplasms in these patients. Patients should be thoroughly informed about the alarm symptoms of malignant neoplasms, especially lymphoma. Cancer in a patient with IEI is more often extensive or disseminated at the time of diagnosis, which is associated with a worse prognosis. Patients with IEI are more likely to develop NHL with B-cell origin, with high histologic grades and extranodal involvement, especially in the gastrointestinal tract or central nervous system. Early diagnosis can provide better treatment options before serious organ damage occurs.

The most prevalent types of malignancies among IEI patients have been summarized in Table 3.

## 6. Diseases of Immune Dysregulation

Diseases of immune dysregulation are a separate and independent category of IEI in IUIS classification [1]. This category includes i.a. familial hemophagocytic lymphohistiocytosis (FHL syndromes), FHL syndromes with hypopigmentation, regulatory T cell defects, autoimmunity with or without lymphoproliferation, immune dysregulation with colitis, ALPS, and a susceptibility to EBV and lymphoproliferative conditions. This category is often the most difficult to define clinically and to diagnose without extensive sequencing since there is a significant phenotypic overlap between different genetic causes, the evolution of features over time, and phenotypic heterogeneity. On the other hand, these diseases have improved our understanding of the pathways that drive autoimmunity in IEI.

Early-onset autoimmunity, autoimmunity that involves multiple organs, a strong family history of autoimmunity, autoimmunity in combination with susceptibility to infection, or significant lymphoproliferation all suggest an immune dysregulation defect.

Diseases of immune dysregulation, according to IUIS classification, are summarized in Table S1.

Over the years, the wide application of whole-exome sequencing/whole-genome sequencing has significantly promoted the discovery and further study of new IEI and its number has doubled from 2009 to 2019 [1,140]. It is worth mentioning that the number of cases for any particular IEI is usually few, and because of that, a large-scale study of IEI can hardly be conducted [140]. Furthermore, there are several difficulties in identifying IEI connected with immune dysregulation. There are still countries where genetic tests are not widespread and freely available, mostly because of their costs. Moreover, in some patients more than one mutation is present, which makes it even more difficult to find [140,141]. In addition, phenotypes of the same mutation vary between patients, ranging from mild or uncharacteristic symptoms to even life-threatening manifestations [140,142,143]. In conclusion, patients with immune dysregulation should be examined scrupulously, and genetic diagnostics should be conducted in cases when it is necessary and possible [140]. Early and proper diagnosis seems crucial when we consider IEI patients. In cases of IEI patients with immune dysregulation, it is even more important.

The treatment is often challenging and sometimes requires balancing between increased susceptibility to infection and the additional suppression of the immune system [144]. Not so long ago, treatment options for IEI patients remained limited. They included the intensive treatment of infections; IRT; and bone marrow transplant in some cases. IRT has been a standard, often live-saving treatment for IEI that has affected antibody production for the past four decades. Both intravenous (IVIg) and subcutaneous (SCIg) immunoglobulins are often suitable for lifelong therapy. High-dose IVIg, together with corticosteroids, is a standard therapy for ITP [144]. A significant increase in the field of clinical immunology, including molecular biology techniques, gene therapy, or the use of immune modulators, allowed the development of modern and precise therapies [145]. Equally, having better knowledge of IEI pathophysiology enables the implementation of targeted therapy. IEI is an excellent example of disease where such “precision medicine” can be applied. Precision medicine is an approach based on advances in genetic research and data analysis. It offers breakthroughs in the treatment of the disease and has the potential to overturn traditional methods of practicing medicine.

Such medicines (new or repurposed) modify intracellular pathways whose function is disturbed because of specific genetic defect [144]. Thanks to precision medicine, the treatment can selectively influence a specific cell function instead of affecting the entire immune system. Moreover, the adverse side effects that affect other tissues are possible to avoid.

Although the term “precision medicine” is relatively new, it has been part of healthcare for many years. For example, a person who needs a blood transfusion does not receive blood from a randomly selected donor; instead, the donor’s blood group is matched to that of the recipient to reduce the risk of complications. Precision medicine is already used in the treatment of diabetes and cancer. It is especially useful in cases of breast, lung, skin, colon, prostate, and pancreatic cancer. Its other promising applications include cardiology, signs of aging, rare childhood diseases, cystic fibrosis, and HIV. 

In the context of immunedysregulation, the usage of small molecules and biologics effectively helps with reversing the clinical manifestations of immunedysregulation and hyperinflammation. Knowledge about the genetic etiology of activated phosphoinositide 3-kinase delta (PI3Kδ) syndrome (APDS) allowed one to explore PI3Kδ inhibition as a precision medicine [146,147]. Leniolisib, a small-molecule, selective PI3Kδ inhibitor, causes the dose-dependent suppression of PI3Kδ pathway hyperactivation. Clinical trials are currently underway to establish the safety and efficacy of selective PI3K δ inhibitors as a possible therapeutic option in patients with APDS. One is related to the oral administration of leniolisib (NCT02435173), the other to the inhaled administration of nemiralisib (NCT02593539). So far, the 12-week dose escalation of leniolisib has been shown to be safe and effective in reducing lymphadenopathy, splenomegaly, and cytopenia [144,147].

## 7. Conclusions

IEI is a group of rare diseases that can be camouflaged or not considered because of the predominant clinical features of atopy, autoimmunity, or lymphoproliferation. Consequently, some patients will remain undiagnosed. This risk impairs their quality of life, morbidity, and mortality, especially when exposed to agents reducing the immune competence. An underlying IEI should be particularly considered, especially in severe cases of atopic disease with concomitant signs of autoimmunity and unusual, recurrent or severe infections, so appropriate treatment regimens can be initiated and inappropriate immune suppression avoided.

In terms of the scientific evidence, it is still debatable whether allergy and cancer should be considered as risk factors or rather the consequences of the underlying IEI. Autoimmunity, as well as malignancy, worsen the IEI patients’ prognosis. Another important issue in IEI is their exact pathogenesis, as well as the gene–phenotype relationship. The recent advances in genetics also revolutionized the field of IEI. Until now, the increased use of new sequencing techniques allowed for the identification of different monogenic causes of IEI. They enabled the better understanding of genotype–phenotype correlations and consequently led to better therapeutic strategies targeting the immune dysregulation in IEI [45]. The unmet needs include the unified nomenclature; the pathophysiological mechanisms assessment, for example, the lymphoma’ genesis in IEI patients; and better, more personalized treatment strategies [148].

Novel diagnostic approaches, as well as evidence-based treatment guidelines that consider the underlying immunodeficiency rather than using extrapolation from non-IEI settings, are necessary. The recommendations for validated screening of cohorts at risk of allergy, autoimmunity, and malignancy are of the utmost importance.

## Figures and Tables

**Table 1:** Inborn errors of immunity with elevated IgE.

Disease	IUIS Classification	Inheritance	Mutation	Characteristics	Immunological Features
Hyper IgE syndrome (HIES)	Combined immunodeficiencies with associated syndromic features	AD LOF	*STAT3*	Infectious disease and immunological manifestations (skin abscesses, recurrent sinopulmonary infections, bacterial infections, pulmonary aspergillus, Pneumocystis jirovecii, and chronic mucocutaneous candidiasis) Craniofacial, dental, musculoskeletal, neurological, and vascular abnormalities	Eosinophilia ↑ IgE ↓-specific antibody production Intermittent chemotactic defectsImpaired inflammatory cytokine production Reduced or absent Th17 cells Defective Th17 cell production of IL-17 Decreased IFN-γ production upon stimulation Decreased CD8^+^ memory T cellsDiminished delayed-type hypersensitivity and lymphoproliferative responses to antigenic stimulation
ZNF341 deficiency (phenocopy of AD-HIES)	Combined immunodeficiencies with associated syndromic features	AR	*ZNF341*	Mild facial dysmorphism Early onset eczema Recurrent bacterial infections (respiratory, skin infections) Lung abscesses and pneumatoceles Musculosceletal abnormalities Retention of primary teeth	↑ IgE- and IgG ↓-specific antibody production ↓ memory B cells excess of Th2 cells ↓ Th17 and NK cells
Loeys–Dietz syndrome (TGFBR deficiency)	Combined immunodeficiencies with associated syndromic features	AD	*TGFBR1* *TGFBR2*	Recurrent respiratory infections Eczema Food allergy Musculosceletal abnormalities Retention of primary teeth Vascular abnormalities	↑ IgE
PGM3 deficiency (hyperimmunoglobulin E-like syndrome with glycosylation defects)	Combined immunodeficiencies with associated syndromic features	AR	*PGM3*	Impaired immunity (recurrent respiratory tract infections, abscesses) Severe atopy, asthma, eczema, and food allergy Autoimmunity Neurocognitive impairment Skeletal dysplasia	Neutropenia T and B cell lymphopenia Eosinophilia ↑ IgE levels N/↑ IgG and IgA Progressive bone marrow failure
Comel–Netherton syndrome	Combined immunodeficiencies with associated syndromic features	AR	*SPINK5*	Congenital ichthyosis Bamboo hair Recurrent bacterial infections Atopy Failure to thrive	↑ IgE and IgA ↓ switched and non-switched B cells
CARD11 deficiency	Combined immunodeficiencies with associated syndromic features	AD LOF	*CARD11*	Severe atopic dermatitis Food allergy Molluscum contagiosum infection Recurrent respiratory infections Lymphoma Various phenotypes from SCID to combined immunodeficiency, associated with atopy and elevated IgE levels or isolated severe atopy	↑ IgE Poor specific antibody production Impaired activation of both NF-kB and mTORC1 pathways N/↓ B cell numbers Defective T-cell activation and proliferation Skewing toward Th2
ERBIN deficiency	Combined immunodeficiencies with associated syndromic features	AD	*ERBB2IP*	Recurrent respiratory infections Susceptibility to S.aureus Eczema Atopy Joint hypermobility, sometimes vascular abnormalities	↑ IgE ↑ circulating Treg
IL6R deficiency	Combined immunodeficiencies with associated syndromic features	AR	*IL6R*	Immunodeficiency (recurrent pyogenic infections, cold abscesses) Atopy Abnormal inflammatory responses	High circulating IL-6 levels Normal/↓ serum IgM, IgG, and IgA Very ↑ IgE ↓-specific antibody productionReduced switched memory B
Interleukin 6 signal transducer (IL6ST) deficiency	Combined immunodeficiencies with associated syndromic features	AR	*IL6ST*	Recurrent infections Boils Eczema Bronchiectasis Pulmonary abscesses Skeletal abnormalities (scoliosis, bone fractures, and craniosynostosis) Retention of primary teeth	Eosinophilia ↑ IgE, Specific antibody production variably affected Impaired B cell memory and acute-phase response ↓ Th17 cells
DOCK8 deficiency	Immunodeficiencies affecting cellular and humoral immunity	AR	*DOCK8*	Recurrent viral and bacterial infections Cutaneous infections (staphylococcal, viral, and fungal) Severe atopy Often multiple severe allergies to food and environmental allergens Hepatic disorders Early-onset malignancy	Eosinophilia ↓ T cell numbers (with normal CD4/CD8 ratio) and variably decreased or normal B- and NK-cell numbers ↓ production of TNFα and IFNγ ↓ numbers of Th17 T cells ↑ Th 2 ↑ IL-4 and IL-13 Few Treg with poor function ↓ IgM levels and variable IgA and IgG levels ↑ IgE Poor antibody responses
TYK2 deficiency	Defects in intrinsic and innate immunity	AR	*TYK2*	Susceptibility to intracellular bacteria (mycobacteria, Salmonella) and viruses Eczema	Impaired cellular responses to IL-10, IL-12, and IL-23 and type I IFNs
Omenn syndrome (OS)	Immunodeficiencies affecting cellular and humoral immunity (usually a T-B-NK^+^ SCID)	AR	various	Erythroderma Alopecia Aplasia/hypoplasia of the eyebrow Desquamation of skin Dry skin Edema Chronic diarrhea Failure to thrive Hepatosplenomegaly Lymphadenopathy Pneumonia Sometimes anemia, autoimmunity, hypothyroidism, and lymphoma	Eosinophilia ↑ IgE Abnormal secretion of IL-4 and IL-5 from activated T cells Exaggerated Th2 response Absence of B cells in the circulation
Wiscott–Aldrich syndrome (WAS)	Combined immunodeficiencies with associated syndromic features	XL	*WAS*	Recurrent bacterial and viral infections Bloody diarrhea Eczema Thrombocytopenia with small platelets ↑ risk of malignancy Autoimmune diseases IgA nephropathy	Eosinophilia Often ↑ IgE and IgA ↓ IgM ↓ antibody responses to polysaccharides Progressive ↓ in T cells numbers Abnormal lymphocyte responses to anti-CD3
Atypical DiGeorge syndrome with deletion of chromosome 22q11.2	Combined immunodeficiencies with associated syndromic features	AD	Deletion typically in chromosome 22	Pharyngeal pouch defects Thymus hypoplasia/aplasia Hypoparathyroidism Congenital heart disease Eczema, erythroderma Lymphadenopathy	Eosinophilia ↑ IgE Partial T cell deficiency Oligoclonal T cells expansion T cell count is higher than typical complete DiGeorge patients
IPEX syndrome (immunodysregulation, polyendocrinopathy, and enteropathy X-linked syndrome)	Diseases of immune dysregulation	XL	*FOXP3*	Multiple endocrinopathies Severe chronic enteropathy Dermatitis Eczema Anemia Thrombocytopenia	↑ IgE and IgA Lack of (and/or impaired function of) CD4^+^ CD25^+^ FOXP3^+^ regulatory T cells (Tregs)

Abbreviations: ↓—decreased; ↑—increased; γ—gamma; AD—autosomal dominant; AR—autosomal recessive; LOF—loss-of-function; N—normal; SCID – severe combined immunodeficiency; Treg—T regulatory cell; and XL—X-linked inheritance.

**Table 2:** Common autoimmune presentation in inborn errors of immunity (IEI).

IUIS Classification	Disease	Main Molecular Defect	Common Autoimmune Disease
Immunodeficiencies affecting cellular and humoral immunity	ICOS deficiency	ICOS	Arthritis, SLE, MS, and enteropathy
Combined immunodeficiencies with associated syndromic features	22q11 deletion syndrome (DiGeorge syndrome)	Large deletion typically in chromosome 22	AIC, AIT, and arthritis
	Wiskott–Aldrich syndrome	WAS	AIC, IBD, GN, arthritis, and vasculitis
Predominantly antibody deficiencies	X-linked agammaglobulinemia	Btk	RA, JIA, IBD, AIC, AIT, PND, KD, DM, T1D, SD, and alopecia
	CVID	Various	AIC (ITP, AIHA, AN), RA, JIA, SLE, IBD, AIT, PA, SS, and vitiligo
	Selective IgA deficiency	Unknown	AIC (ITP, AIHA), IBD, CD, PV, MG, SLE, RA, JIA, T1D, and AIT
	P110 delta deficiency	PIK3CD	IBD, AIC
	Hyper IgM syndrome	CD40, CD40L	AIT, IBD, RA, JIA, AIHA, and AGN
Diseases of immune dysregulation	LRBA deficiency	LRBA	AIC (AIHA, ITP, AN), IBD, RA, and JIA
	APECED	AIRE	T1D, AD, AIT, hypoparathyroidism, enteropathy, adrenal corticotropic hormone insufficiency, growth hormone insufficiency, vitiligo, alopecia, autoimmune hepatitis, and ovarian/testicular failure
	IPEX	FOXP3	IBD, AIC, AIT, vitiligo, alopecia, hepatitis, and early onset diabetes
	CTLA4 haploinsufficiency	CTLA4	IBD, AIC, SLE, and arthritis
	XIAP deficiency	XIAP	IBD, AIC, and hepatitis
	Early onset inflammatory bowel disease syndromes	various	IBD, arthritis
	STAT3 GOF	STAT3	IBD, AIC, hepatitis, and early-onset T1D
	ALPS	various	AIC, GN, endocrinopathies, and SLE
Congenital defects of phagocyte number, function, or both	Chronic granulomatous disease	CYBB	IBD, AIC, AIT, JIA, GN, SLE, APLA, and autoimmune pulmonary disease
Defects in innate immunity	STAT1 deficiency	STAT1 GOF	AIC, AIT, T1D, and SLE
Autoinflammatory disorders	Type 1 interferonopathies	various	SLE, AIC, and vasculopathy
Complement deficiencies	Complement deficiencies	various	SLE, vasculitis

Abbreviations: AD—Addison’s disease; AIC—autoimmune cytopenia; AIHA—autoimmune hemolytic anemia; AIT—autoimmune thyroid disease; AN—autoimmune neutropenia; ALPS—autoimmune lymphoproliferative syndrome; APECED—autoimmune polyendocrinopathy-candidiasis-ectodermal dystrophy; APLA—antiphospholipid antibodies; CD—celiac disease; CVID—common variable immunodeficiency; GN—glomerulonephritis; GOF—gain-of-function; IBD—inflammatory bowel disease; IUIS—International Union of Immunological Societies; JIA—juvenile idiopathic arthritis; IPEX—immune dysregulation, polyendocrinopathy, enteropathy, and X-linked syndrome; ITP—immune thrombocytopenia; MS – multiple sclerosis; RA—rheumatoid arthritis; SLE—systemic lupus erythematosus; and T1D—type 1 diabetes.

**Table 3:** Most common types of cancer among patients with IEI.

Disease	IUIS Classification	Type of Malignancy
SCID	Immunodeficiencies affecting cellular and humoral immunity (Ia)	Lymphoma
ITK deficiency	Immunodeficiencies affecting cellular and humoral immunity (Ib)	EBV-associated lymphoproliferation Lymphoma
IKAROS deficiency (CD154)	Immunodeficiencies affecting cellular and humoral immunity (Ib)	T-ALL
DOCK8 deficiency	Immunodeficiencies affecting cellular and humoral immunity (Ib)	Vulvar, facial, and anal squamous cell dysplasia and carcinomas;T cell lymphoma-leukemiaBurkitt lymphomaNHL
STK4 deficiency	Immunodeficiencies affecting cellular and humoral immunity (Ib)	Lymphoma
RHOH deficiency	Immunodeficiencies affecting cellular and humoral immunity (Ib)	Lymphoma
OX40 deficiency	Immunodeficiencies affecting cellular and humoral immunity (Ib)	Kaposi sarcoma
CD40/CD40L deficiency	Immunodeficiencies affecting cellular and humoral immunity (Ib)	Hepatocarcinoma Cholangiocarcinoma Peripheral neuroectodermal tumors of the gastrointestinal tract and the pancreas Lymphoma
Wiskott–Aldrich syndrome	Combined immunodeficiency of T and B cell with associated or syndromic features	Lymphoma EBV-related B-cell lymphoma Leukemia Cerebellar astrocytoma Kaposi sarcoma Smooth muscle tumors
Ataxia-telangiectasia	Combined immunodeficiency of T and B cell with associated or syndromic features	Leukemia Lymphoma Breast cancer Gastrointestinal malignancies (possible)
Nijmegen breakage syndrome	Combined immunodeficiency of T and B cell with associated or syndromic features	Lymphoma Acute leukemia Solid tumors
Bloom syndrome	Combined immunodeficiency of T and B cell with associated or syndromic features	Leukemia Lymphoma
PMS2 deficiency	Combined immunodeficiency of T and B cell with associated or syndromic features	Lymphoma Colorectal carcinoma Brain tumors
MCM4 deficiency	Combined immunodeficiency of T and B cell with associated or syndromic features	B cells lymphoma
Ligase I deficiency	Combined immunodeficiency of T and B cell with associated or syndromic features	Lymphoma
Cartilage-hair hypoplasia	Combined immunodeficiency of T and B cell with associated or syndromic features	Lymphoma Leukemia Squamous cell carcinoma Basal cell carcinoma
Schimke syndrome	Combined immunodeficiency of T and B cell with associated or syndromic features	Osteosarcoma NHL
Autosomal dominant hyper-IgE syndrome (AD-HIES)	Combined immunodeficiency of T and B cell with associated or syndromic features	NHL
CID with early-onset asthma, eczema and food allergies, autoimmunity ID with atopic dermatitis (CARD11)	Combined immunodeficiency of T and B cell with associated or syndromic features	Lymphoma
X-linked agammaglobulinemia	Predominantly antibody deficiencies	Lymphoreticular malignancies Gastric and colorectal adenocarcinoma Squamous cell carcinoma of the lung
Common variable immunodeficiency (CVID)	Predominantly antibody deficiencies	Lymphoma Thymus cancer Gastric cancer
Selective IgA deficiency	Predominantly antibody deficiencies	Gastrointestinal cancer
X-linked lymphoproliferative disease (XLP1)	Diseases of immune dysregulation	Lymphoma
CD27 deficiency	Diseases of immune dysregulation	Lymphoma
RASGRP1 deficiency	Diseases of immune dysregulation	EBV-associated lymphoma
CD70 deficiency	Diseases of immune dysregulation	Hodgkin lymphoma
CTPS1 deficiency	Diseases of immune dysregulation	B-cell NH lymphoma
CD137 deficiency	Diseases of immune dysregulation	B-cell lymphoma
XL magnesium EBV and neoplasia (XMEN)	Diseases of immune dysregulation	Lymphoma
ALPS–FAS	Diseases of immune dysregulation	Lymphoma
Severe congenital neutropenia	Congenital defects of phagocyte number, function, or both	MDS/leukemia
HAX1 deficiency	Congenital defects of phagocyte number, function, or both	MDS/leukemia
Shwachman-Diamond syndrome	Congenital defects of phagocyte number, function, or both	Leukemia
GATA2 deficiency	Congenital defects of phagocyte number, function, or both	AML/CMML
WHIM syndrome	Defects in intrinsic and innate immunity	HPV-related cancers Lymphoma
Epidermodysplasia verruciformis	Defects in intrinsic and innate immunity	Squamous cell carcinoma

Abbreviations: AML—acute myelogenous leukemia; CMML—chronic myelomonocytic leukemia; EBV—Epstein–Barr virus; HPV—human papillomavirus; MDS—myelodysplastic syndrome; NHL—non-Hodgkin lymphoma; and T-ALL—T-cell acute lymphoblastic leukemia.

## Data Availability

Not applicable.

## Supplementary Materials

The following supporting information can be downloaded at: https://www.mdpi.com/article/10.3390/jcm11144220/s1, Table S1: Diseases of immune dysregulation according to IUIS classification. Accessed on 8 July 2022.
