# Supplementary material for: Diagnostic Challenges in Patients with Inborn Errors of Immunity with Different Manifestations of Immune Dysregulation
Source: J Clin Med. 2022 Jul 20;11(14):4220. doi: 10.3390/jcm11144220 (PMC9324612; doi:10.3390/jcm11144220)
Supplement: Supplementary file 1 [file jcm-11-04220-s001.zip › jcm-1808503-supplementary.pdf]

Table S1. Diseases of immune dysregulation according to IUIS classification (2022). Accesed on 8th July 2022.

Category	Disease	Genetic Defect	Clinical Characteristics
Familial Hemophagocytic Lymphohistiocytosis (FHL syndromes)	Perforin deficiency (FHL2)	*PRF1*	Fever<br>Hepatosplenomegaly<br>Hemophagocytic lymphohistiocytosis<br>Cytopenias
	UNC13D/Munc13-4 deficiency (FHL3)	*UNC13D*	Fever<br>Hepatosplenomegaly<br>Hemophagocytic lymphohistiocytosis<br>Cytopenias
	Syntaxin 11 deficiency (FHL4)	*STX11*	
	STXBP2/ Munc18-2 deficiency (FHL5)	*STXBP2*	
	FAAP24 deficiency	*FAAP24*	EBV-driven lymphoproliferative disease
	SLC7A7 deficiency	*SLC7A7*	Lysinuric protein intolerance<br>Bleeding tendency<br>Alveolar proteinosis
	RHOG deficiency	*RHOG*	Hemophagocytosis<br>Fever<br>Hepatosplenomegaly<br>Cytopenias<br>Low haemoglobin<br>Hypertriglyceridemia<br>Elevated ferritin<br>sCD25
FHL syndromes with hypopigmentation	Chediak-Higashi syndrome	*LYST*	Fever<br>Hepatosplenomegaly<br>Hemophagocytic lymphohistiocytosis<br>Partial albinism<br>Recurrent infections<br>Progressive neurological dysfunction<br>Cytopenias<br>Neutropenia<br>Bleeding tendency<br>Giant lysosomes
	Griscelli syndrome type 2	*RAB27A*	Fever<br>Hepatosplenomegaly<br>Hemophagocytic lymphohistiocytosis<br>Partial albinism<br>Cytopenias
	Hermansky-Pudlak syndrome type 2	*AP3B1*	Hemophagocytic lymphohistiocytosis<br>Partial albinism<br>Recurrent infections<br>Pulmonary fibrosis<br>Neutropenia<br>Bleeding tendency
	Hermansky-Pudlak syndrome type 10	*AP3D1*	Recurrent infections<br>Oculocutaneous albinism<br>Seizures<br>Hearing loss<br>Neurodevelopmental delay<br>Neutropenia
	CEBPE neofunction	*CEBPE*	Recurrent abdominal pain<br>Aseptic fever<br>Systemic inflammation<br>Abscesses<br>Ulceration<br>Infections<br>Mild bleeding diathesis
Regulatory T cells defects	IPEX syndrome (immune dysregulation, polyendocrinopathy, enteropathy X-linked)	*FOXP3*	Autoimmune enteropathy<br>Early onset diabetes<br>Thyroiditis<br>Eczema<br>Hemolytic anemia

			Thrombocytopenia<br>↑IgE, IgA<br>Lack of CD4+CD25+FOXP3+ regulatory T cells (Tregs)
	CD25 deficiency	*IL2RA*	Lymphoproliferation<br>Autoimmunity<br>Impaired T cell proliferation in vitro
	CD122 deficiency	*IL2RB*	Recurrent viral infections<br>Lymphoproliferation<br>Hepatosplenomegaly<br>Lymphadenopathy<br>Enteropathy<br>Dermatitis<br>Autoimmune hemolytic anemia<br>Hypergammaglobulinemia
	CTLA4 haploinsufficiency (ALPS-V)	*CTLA4*	Recurrent infections<br>Enteropathy<br>Interstitial lung disease<br>Autoimmune cytopenias<br>Extra-lymphoid lymphocytic infiltration
	LRBA deficiency	*LRBA*	Recurrent infections<br>Autoimmunity<br>Inflammatory bowel disease
	DEF6 deficiency	*DEF6*	Recurrent infections<br>Enteropathy<br>Cardiomyopathy<br>Hepatosplenomegaly
	STAT3 GOF	*STAT3*	Recurrent infections<br>Lymphoproliferation<br>Solid organ autoimmunity
	BACH2 deficiency	*BACH2*	Sinopulmonary infections<br>Lymphocytic colitis
	FERMT1 deficiency	*FERMT1*	Dermatosis<br>Photosensitivity<br>Skin atrophy<br>Skin fragility<br>Scaling
	IKAROS GOF	*IKZF1*	Recurrent infections<br>Multiple autoimmune features:diabetes, colitis, thyroiditis<br>Allergy<br>Lymphoproliferation<br>Plasma cell expansion<br>Evans syndrome
Autoimmunity with or without lymphoproliferation	APECED (APS-1; autoimmune polyendocrinopathy with candidiasis and ectodermal dystrophy)	*AIRE*	Autoimmune diseases: hypoparathyroidism, hypothyroidism, adrenal insufficiency, diabetes, gonadal dysfunction and other<br>Alopecia areata<br>Enteropathy<br>Dental enamel hypoplasia<br>Pernicious anemia<br>Chronic mucocutaneous candidiasis
	ITCH deficiency	*ITCH*	Autoimmunity (thyroiditis, diabetes type I, hepatitis, enteropathy)<br>Early-onset chronic lung disease<br>Failure to thrive<br>Developmental delay<br>Dysmorphic facial features
	Tripeptidyl-Peptidase II deficiency	*TPP2*	Recurrent infections<br>Lymphoproliferation<br>Severe autoimmune cytopenias<br>Hypergammaglobulinemia
	JAK1 GOF	*JAK1*	Hepatosplenomegaly<br>Viral infections<br>Poor growth<br>Thyroid disease<br>Eosinophilic enteritis
	Prolidase deficiency	*PEPD*	Infections

			Eczema<br>Chronic skin ulcers<br>Autoantibodies
	SOCS1 haploinsufficiency	*SOCS1*	Recurrent bacterial infections<br>Early onset severe multisystemic autoimmunity<br>Arthritis, thyroiditis, hepatitis, psoriasis<br>Hepatosplenomegaly<br>Systemic lupus erythematosus<br>Glomerulonephritis<br>Cytopenias
	PD-1 deficiency	*PDCD1*	Hepatosplenomegaly<br>Autoimmunity (type 1 diabetes, hypothyroidism, juvenile idiopathic arthritis)<br>Fatal pulmonary autoimmunity<br>Tuberculosis
Immune dysregulation with colitis	IL-10 deficiency	*IL10*	Recurrent respiratory diseases<br>Folliculitis<br>Arthritis<br>Inflammatory bowel disease
	IL-10R deficiency	*IL10RA/ IL10RB*	Recurrent respiratory diseases<br>Folliculitis<br>Arthritis<br>Inflammatory bowel disease<br>Lymphoma
	NFAT5 haploinsufficiency	*NFAT5*	Inflammatory bowel disease<br>Recurrent sinopulmonary infections
	TGFB1 deficiency	*TGFB1*	Inflammatory bowel disease<br>Recurrent viral infections<br>Microcephaly<br>Encephalopathy
	RIPK1	*RIPK1*	Early onset inflammatory bowel disease<br>Recurrent infections<br>Progressive polyartritis
	ELF4 deficiency	*ELF4*	Early onset inflammatory bowel disease/mucosal autoinflammation<br>Fevers<br>Ulcers
Autoimmune Lymphoproliferative Syndrome (ALPS)	ALPS-FAS	*TNFRSF6*	Splenomegaly<br>Adenopathies<br>Autoimmune cytopenias<br>↑ lymphoma risk<br>↑serum FasL<br>N/↑IgG,IgA
	ALPS-FASLG	*TNFSF6*	Splenomegaly<br>Adenopathies<br>Autoimmune cytopenias<br>SLE<br>Soluble FasL is not elevated
	ALPS- Caspase 10	*CASP10*	Splenomegaly<br>Adenopathies<br>Autoimmunity
	ALPS- Caspase 8	*CASP8*	Splenomegaly<br>Adenopathies<br>Viral and bacterial infections<br>Hypogammaglobulinemia
	FADD deficiency	*FADD*	Viral and bacterial infections<br>Functional hyposplenism<br>Recurrent episodes of encephalopathy and liver dysfunction
Susceptibility to EBV and lymphoproliferative conditions	SAP deficiency (XLP1)	*SH2D1A*	Clinical and immunologic features triggered by EBV infection:<br>Hemophagocytic lymphohistiocytosis<br>Lymphoproliferation<br>Aplastic anemia<br>Lymphoma<br>Hypogammaglobulinemia
	XIAP deficiency (XLP2)	*XIAP*	EBV infection<br>Lymphoproliferation

		Splenomegaly<br>Hemophagocytic lymphohistiocytosis<br>Colitis<br>Inflammatory bowel disease<br>Hepatitis
CD27 deficiency	*CD27*	Features triggered by EBV infection:<br>Hemophagocytic lymphohistiocytosis<br>Aplastic anemia<br>B-Lymphoma
CD70 deficiency	*CD70*	EBV susceptibility<br>Hodgkin lymphoma<br>Autoimmunity
CTPS1 deficiency	*CTPS1*	Recurrent/chronic bacterial or viral infections<br>EBV lymphoproliferation<br>B-cell non-Hodgkin lymphoma
CD137 deficiency	*TNFRSF9*	EBV lymphoprolypheration<br>Chronic active EBV infection<br>B-cell lymphoma<br>↓IgG, IgA
RASGRP1	*RASGRP1*	Recurrent pneumonia<br>Herpesvirus infections<br>EBV associated lymphoma<br>↓NK function
RLTPR deficiency	*CARMIL2*	Recurrent bacterial, fungal and mycobacterial infections<br>Viral warts<br>Malignancy<br>Atopy
X-linked magnesium EBV and neoplasia (XMEN)	*MAGT1*	EBV infection<br>Viral infection<br>Respiratory and gastrointestinal infections<br>Lymphoma<br>Glycosylation defects
PRKCD deficiency	*PRKCD*	EBV chronic infection<br>Recurrent infections<br>Lymphoproliferation<br>SLE-like autoimmunity<br>Low IgG
TET2 deficiency	*TET2*	ALPS-like, recurrent viral infections<br>EBV viremia<br>Lymphadenopathy<br>Hepatosplenomegaly<br>Autoimmunity<br>B-cell lymphoma<br>Developmental delay
